# Relationship between Handgrip Strength and Muscle Mass in Female Survivors of Breast Cancer: A Mediation Analysis

**DOI:** 10.3390/nu9070695

**Published:** 2017-07-04

**Authors:** Lorena Benavides-Rodríguez, Antonio García-Hermoso, Diogo Rodrigues-Bezerra, Mikel Izquierdo, Jorge Enrique Correa-Bautista, Robinson Ramírez-Vélez

**Affiliations:** 1 Centro de Estudios para la Medición de la Actividad Física (CEMA), Escuela de Medicina y Ciencias de la Salud, Universidad del Rosario, Bogotá DC 111221, Colombia; lorena.benavides.rodriguez@gmail.com (L.B.-R.); jorge.correa@urosario.edu.co (J.E.C.-B.); 2 Laboratorio de Ciencias de la Actividad Física, el Deporte y la Salud, Facultad de Ciencias Médicas, Universidad de Santiago de Chile, USACH, Región Metropolitana, Santiago 7500618, Chile; antonio.garcia.h@usach.cl; 3 Grupo GICAEDS, Facultad de Cultura Física, Deporte y Recreación, Universidad Santo Tomás, Bogotá DC 110311, Colombia; diogobezerra11@gmail.com or diogorodrigues@usantotomas.edu.co; 4 Department of Health Sciences, Public University of Navarre, CIBER de Fragilidad y Envejecimiento Saludable (CB16/10/00315), Tudela, Navarre 31006, Spain; mikel.izquierdo@gmail.com

**Keywords:** prevalence, body composition, sarcopenia, breast cancer, physical performance, adults

## Abstract

This study explored the mediating factors of sarcopenia in a group of women survivors of breast cancer in Bogotá, Colombia. This was a descriptive cross-sectional study with 98 women survivors of breast cancer, who were registered with the SIMMON (Integrated Synergies to Improve Oncological Management in Colombia) Foundation. Body weight, height, and waist circumference (WC) were measured, and body mass index (BMI) was calculated. Body composition (percentage of fat and muscle mass) was evaluated via four-pole bioelectrical impedance analysis. Sarcopenia was defined as low muscle mass plus low grip strength or low gait speed (European Working Group on Sarcopenia in Older People (EWGSOP) criteria). A “causal” mediation analysis with the Baron & Kenny procedure (PROCESS^®^ macro, Columbus, OH, USA) was used to explore variables related to sarcopenia. Analyses were performed with the IBM SPSS 21 statistical package (SPSS Inc., Chicago, IL, USA). The significance level of the results obtained in the hypothesis contrast was *p* < 0.05. The mean age of the sample was 65.5 ± 5.9 years, with a BMI of 27.8 ± 4.7 kg/m^2^. The prevalence of sarcopenia was 22.4%. Linear regression models suggest a partial mediation of anthropometric parameters (body mass, body mass index and waist circumference) in the association between handgrip strength and muscle mass. In conclusion, one in every five women survivors of breast cancer had sarcopenia. The findings seem to emphasize the importance of obesity prevention in women survivors of breast cancer, suggesting that high handgrip strength may not relate closely to greater muscle mass and therefore would not exclude the risk of sarcopenia.

## 1. Introduction

Cancer is one of the main causes of death on a global scale. In 2012, 8.2 million registered deaths were attributed to this cause [1]. In Colombia, according to statistics presented by the 2012 GLOBOCAN project (International Agency for Research on Cancer) of the World Health Organization (WHO), approximately 104 people died each day due to this disease and 196 people contracted cancer each day [2]. An analysis from the Sub-direction for Non-communicable Diseases at the Ministry of Health and Social Protection shows that breast cancer in Colombia is classified as a public health problem. It is estimated that nearly 8600 cases are detected per year, and the highest numbers of new cases are registered in the cities of Bogotá, Medellín, Cali, Barranquilla, Cartagena, Bucaramanga, Santa Marta, and San Andrés [3]. Likewise, the World Health Organization reports that breast cancer is the most frequent type of cancer among women, representing 16% of all cancers in women. In Colombia, breast cancer shows a higher mortality rate than any other clinical entity (approximately 2649 deaths/year for every 100,000 inhabitants) [3].

Recent scientific progress in cancer screening, diagnosis, and treatment translates into a greater prevalence of cancer and an increased number of survivors [1,2]. In addition, the treatments that women undergo after being diagnosed with breast cancer produce adverse changes, principally in their quality of life and functional capacity [4]. One factor that seems to potentially produce a significant reduction in morbidity and mortality in survivors of any type of cancer is maintenance of adequate body weight, especially in relation to body composition (muscle tissue and fat distribution) [5]. Regarding body composition, people who endure neoplastic diseases show lower lean body mass, with conserved or increased percentages of fat [6]. Thus, the relationship that is frequently observed between reduced muscle mass and strength is often independent of the proportion and distribution of adipose tissue [7,8].

In addition, sarcopenia*—*a condition characterized by loss of skeletal muscle mass and function—is also related to mobility disorders, a higher risk of falls and fractures, deterioration of the capacity to perform everyday activities, disability, loss of independence, and a greater risk of death [9]. It has been reported that another frequent and persistent problem for many breast cancer survivors is increased weight. In obese cancer patients who lose muscle mass, this culminates in sarcopenic obesity (SO), a condition that may be masked by the excess fat mass [10]. SO was defined for the first time in 1996 as the combination of reduced fat-free mass and excess fat mass, evaluated by bioimpedance analysis (BIA) and expressed as body weight percentage [6]. Low muscle density, low muscle strength and a higher proportion of fat infiltration within muscle have recently been recognized as additional features of sarcopenia along with muscle depletion [10,11].

Additionally, observational studies show that the side effects of cancer treatment can include a metabolic state that induces muscular dysfunction, sarcopenia, and SO [12,13]. In breast cancer survivors, some non-conclusive evidence seems to exist that mortality for overweight and obese women is worse than for women with normal weight, even with the same diagnosis. One possible reason for this association is that comorbidity might be more common in sarcopenic patients as a result of potentially decreased overall health status [14]. Another explanation may be due to the pharmacokinetics of cytotoxic drugs; individual variations in cancer patients may cause the drug distribution volume to vary up to threefold in relation to the corresponding body surface area, on which the dosage conventionally is determined [10]. Thus, SO patients may have a higher susceptibility to toxicity compared to obese patients with a normal lean body mass [10]. Although the exact mediating factors in relation to sarcopenia and SO are not fully understood, these body composition types have been associated with cancer complications.

Although sarcopenia is a problem affecting public health and the quality of life of patients who have completed breast cancer treatment, few studies in Colombia have addressed its prevalence or the factors that can be related to this clinical manifestation. The present study focuses on determining the mediating factors of sarcopenia in a group of women who survived breast cancer in Bogotá, Colombia.

## 2. Methods

### 2.1. Study Design and Sample Population

During 2015 and 2016, a cross-sectional study was conducted on 98 women over 60 years of age who lived in the city of Bogotá and were registered with the SIMMON Foundation. The study recruited women “cancer survivors”—defined in 2011 by the Centers for Disease Control and Prevention as those individuals who have been diagnosed with cancer, from the moment of the diagnosis to the rest of their lives—ranging in age from 60 to 75 years and residing in the urban zone of the city of Bogotá, Colombia. The study excluded women with acute conditions or chronic acutely decompensated conditions, pacemaker-type devices, terminal chronic kidney disease being treated by hemodialysis, or musculoskeletal diseases that limit implementation of tests.

### 2.2. Data Collection

Participants who accepted and signed the informed consent form provided information about their clinical history, indicating personal and family disease antecedents, sociodemographic aspects, education and socioeconomic level, and other information. The anthropometric evaluation included measurements of height registered in a stretched position with portable stadiometer (Seca 206^®^; Hamburg, Germany) (range 0–220 cm) with 1 mm precision. Weight was measured with a Tanita floor scale (Seca mBCA 515^®^, HANS E. RÜTH S.A., Hamburg, Germany) with a maximum capacity of 200 kg and minimum capacity of 100 g. These variables were used to calculate the body mass index (BMI) in kg/m^2^, adopting the cut-off recommended by the World Health Organization. Thereafter, waist circumference (WC) was measured with using a plastic measuring tape with 1 mm precision (Holtain Ltd., Crymych Dyfed, UK), using the anatomic reference points described by the World Health Organization. Evaluation of body composition (fat mass and muscle mass) was conducted via tetrapolar bioelectrical impedance analysis (BIA) (Seca mBCA 515^®^, HANS E. RÜTH S.A., Hamburg, Germany) by following the indications and equations indicated in the user manual. Induction frequency was evaluated at an intensity of 50 kHz, with 0.1 kg (0.1%) fat mass estimation sensitivity. This analyzer allowed us to ascertain fat mass (% and kg), muscle mass (% and kg), musculoskeletal mass (kg), and skeletal muscle mass index (kg/m^2^). No patients had clinically detectable edema, a condition that could have affected resistance and reactance. This measurement was carried out with an empty bladder and on a non-conducting stable surface, following 10–12 h of fasting.

For sarcopenia diagnosis, the study followed the guidelines furnished by the European consensus for its definition and diagnosis (European Working Group on Sarcopenia in Older People (EWGSOP)) [15]. These guidelines included the confirmation of criteria to classify the population according to the stage of sarcopenia, with the first criterion referring to low muscle mass, the second criterion related to low muscle strength, and the third criterion based on low physical performance. To be diagnosed with sarcopenia, a woman needed to fulfill criterion number 1 in addition to criterion 2 or 3 (Table 1).

The skeletal muscle mass index (criterion of muscle mass) was determined through tetrapolar BIA (Seca mBCA 515^®^, HANS E. RÜTH S.A., Hamburg, Germany) [15]. For muscle strength, handgrip strength was measured with a digital dynamometer (0.1 kg precision by Takei Smedley III T-19^®^, Scientific Instruments Co. Ltd., Niigata, Japan). Researchers provided standardized verbal instructions describing the correct execution of the test before providing a visual demonstration of the technique. Dynamometers were adjusted to account for differences in hand size. Handgrip strength was measured with the subject in a standing position with the shoulder adducted and neutrally rotated and arms parallel but not in contact with the body. The participants were asked to squeeze the handle for a maximum of 3–5 s. Verbal encouragement was given during the test. Participants performed two trials with each hand. The values used in the present study represent the highest value obtained with either hand. For physical performance, the study applied the Short Physical Performance Battery by following the protocol from the validation for Colombia [16]. This study evaluated standing from a chair tests (registering the number of repetitions), equilibrium (time in s) and gait speed (m/s). For the standing test from a chair, participants were asked to stand and sit in a chair five times as quickly as they could with arms crossed over the chest. This test was performed only after the participant demonstrated their ability to stand without using their arms. For equilibrium, participants were asked to remain standing with their feet as close together as possible, then in a semi-tandem position (the ankle of one foot behind the joint of the other foot and finally in a tandem position (ankle of one foot directly behind the other foot and touching it) [16]. Each position had to be held for 10 s. The gait speed was evaluated by asking participants to walk 10 m distance. Time was recorded from the 2 m to 8 m marker. Then, the gait speed was calculated from time using in 6 m walk with the cut-off value of 0.8 m/s. Gait seep was obtained dividing the distance by the time and it was reported in meters per second (m/s) [16].

To establish “severe sarcopenia”, women were required to present low muscle mass <6.42 kg/m^2^ in the skeletal muscle mass index, low muscle strength <20 kg in the handgrip strength test, and ≤0.8 m/s in the gait speed test, as indicator of low physical performance [15]. For the stage of “sarcopenia”, women had to have low muscle mass, along with low muscle strength or deficient physical performance. Lastly, women with low muscle mass, but without changes in muscle strength or physical performance were classified in the “pre-sarcopenia” stage. Women who only had low values of muscle strength according to the handgrip test, but adequate values of muscle mass and physical performance were classified at “risk of sarcopenia”. Finally, to define SO, we applied the equation used by Kim et al. [17] which required the participant to have a muscle mass percentage <30.7% of the total weight and fat mass percentage >31.7%, determined through tetrapolar BIA (Seca mBCA 515^®^, HANS E. RÜTH S.A, Hamburg, Germany).

### 2.3. Ethics Statement

The study was conducted in accordance with the Helsinki Declaration for Human Studies and approved by the Colombian Data Protection Authority (Resolution 008430/1993 Ministry of Health) and the Review Committee for Research on Human Subjects at the University of Rosario (Code CEI-ABN026-000173). All participants were informed of the study’s goals, and written informed consent was obtained.

### 2.4. Statistical Analysis

Information was processed and analyzed with the Statistical Package for Social Science^®^ software, version 21 (SPSS Inc., Chicago, IL, USA). The normality of the distribution of the variables was tested using the Kolmogorov-Smirnov test. Characteristics of body composition and physical condition of the study were presented with mean values, standard deviation (SD), and frequencies. Prevalence, expressed in proportions, was calculated according to the number of patients who had one or more criteria for diagnosis of sarcopenia or SO. Differences between the categories of sarcopenia and the variables studied were analyzed through analysis of variance (ANOVA) in the continuous variables and the linear chi squared test on variables of proportion. To examine whether the association between handgrip strength and muscle mass was mediated by anthropometric parameters, linear regression models were fitted using bootstrapped mediation procedures included in the PROCESS SPSS macro [18]. The test of multicollinearity was done before analyzing mediation models due to relationship between dependent and mediators variables. The tolerance value and variance inflation factors value appeared normal with values ranging between 0.119 and 0.446 [19] for collinearity tolerance and 2.244 and 8.396 for collinearity [20]. The results were statistically significant with *p* < 0.05.

## 3. Results

### 3.1. Descriptive Characteristics

Sociodemographic variables, body composition, and diagnostic criteria for sarcopenia are shown in Table 2. In the entire sample, the mean age was 65.53 ± 5.91 years, weight was 63.95 ± 11.3 (kg), BMI was 27.88 ± 4.71 kg/m^2^, skeletal muscle mass index was 7.31 ± 1.07 kg/m^2^, handgrip strength was 16.79 ± 4.98 kg, and physical performance evaluated through gait speed was 1.22 ± 0.40 m/s. The prevalence rates of sarcopenia and SO was were 22.4% and 16.3%, respectively.

### 3.2. Body Composition and Sarcopenia Stages from the EWGSOP

Table 3 presents the sample distribution according to the stages proposed in this work (healthy, risk of sarcopenia, presarcopenia, sarcopenia, severe sarcopenia) and the variables studied. In the absence of sarcopenia (healthy/risk of sarcopenia), significantly lower values were observed in the anthropometric variables (weight, WC, and fat mass).

### 3.3. Body Composition and Mediation Analysis

**Figure 1:**
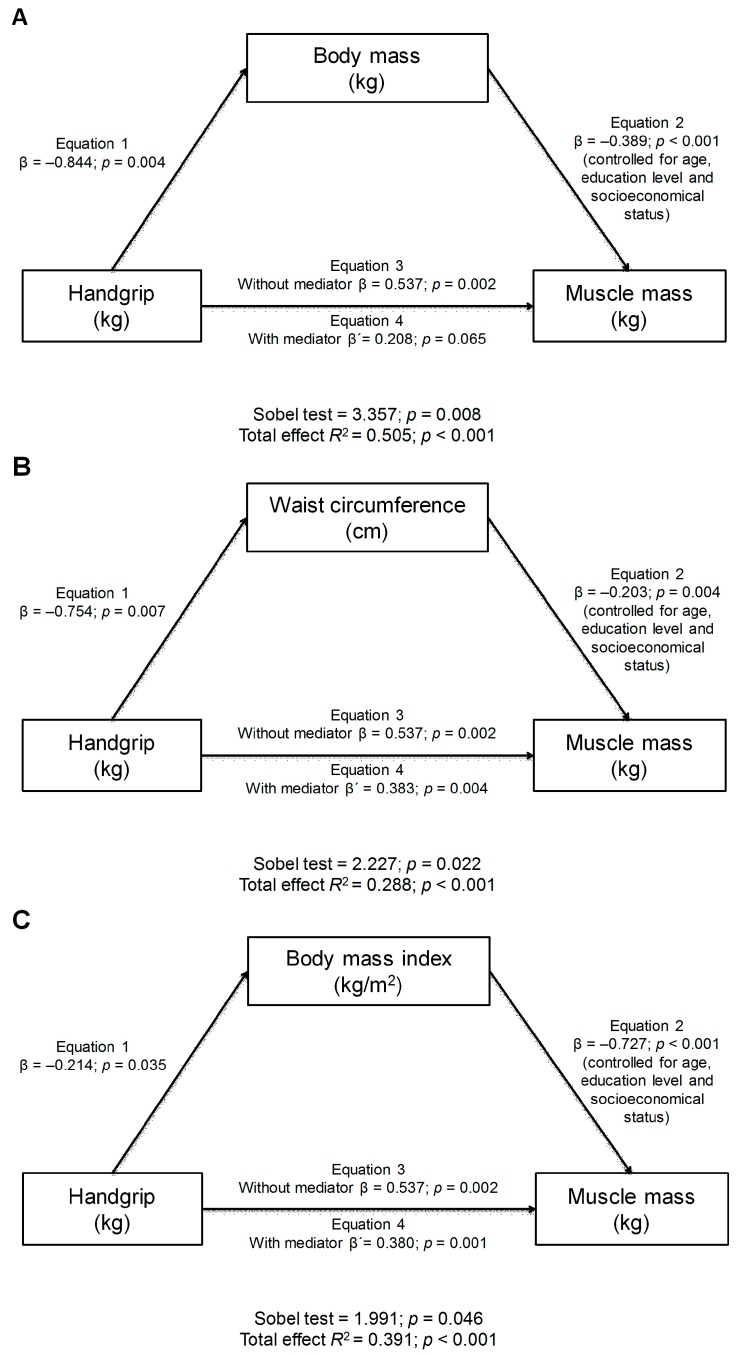
Anthropometric mediation models of the relationship between handgrip strength and muscle mass, adjusted by age, education level, and socioeconomic status. (**A**): body mass; (**B**): waist circumference; (**C**): body mass index; β: unstandardized regression coefficient.

We performed a mediation analysis to test whether anthropometric parameters acted as mediator variables between muscle mass (dependent variable) and handgrip strength (independent variable) (Figure 1). In the first regression (Equation (1)), handgrip strength was negatively associated with the anthropometric parameters (body mass, BMI and WC) (*p* < 0.01). In the second regression (Equation (2)), handgrip strength was positively associated (*p* = 0.002) with the dependent variable (muscle mass). In the last regression model, the mediator variable was negatively associated with the dependent variable (Equation (3)) (*p* < 0.01), but when anthropometric parameters (body mass, BMI and WC) were included in the model (Equation (4)), the regression coefficient did not maintain its statistical significance (full mediation) with body mass (*p* = 0.065) but did so with BMI and WC (partial mediation).

## 4. Discussion

As far as we know, our study is the first to evaluate the mediating role of anthropometric parameters (i.e., body mass, BMI and WC) in the relationship between handgrip strength and muscle mass, the pivotal parameter in the definition of sarcopenia [15,21]. In our sample, approximately one in every five women who had survived breast cancer had sarcopenia. Mediation analysis seems to emphasize the importance of obesity prevention in women survivors of breast cancer, suggesting that high levels of handgrip strength may not be closely related to greater muscle mass. In this line, the potential role of behavioral interventions, nutritional changes, and pharmacological strategies to attenuate obesity-related inflammation is a key research objective.

Using the criteria defined by the EWGSOP, this study detects a 22.4% prevalence of sarcopenia, a higher prevalence than was found in studies published by Baumgartner et al. [22] (13%), Melton et al. [23] (8%), Villasenor et al. [14] (15%), and Yamada et al. [24] (11.5%) even in older adults from Bogotá (13.8%) [25]. Other published studies, which use bioelectrical impedance to measure muscle mass and apply the EWGSOP criteria for sarcopenia diagnosis, also yield results quite different from those found in this work. For example, a reanalysis of the study by InCHIANTI Study (Invecchiare in Chianti, aging in the Chianti area) [26] in a population of 538 elderly Italian adults, using the muscle mass cut-off recommended in the EWGSOP consensus, found that the prevalence of sarcopenia in the age range of 70 to 74 years was 1.2% in men and 2.6% in women. Our study also reported that the prevalence for risk of sarcopenia was 46.3%, which could precede to sarcopenia due to women were classified as weak, assessed with the handgrip test. Low levels of muscular strength are an important indicator that helps identify the level of development and degree of disability. For example, muscle strength is associated with a number of causes resulting in cardiovascular death [27], in addition to bone fragility, and the presence of sarcopenia [28]. Furthermore, low levels of strength are also associated with mobility disorders, increased risk of falls, reduced ability to function in daily living activities, loss of independence, and reduced life expectancy [29]. Therefore, the European Society for Clinical Nutrition and Metabolism redefined sarcopenia as a condition characterized by loss of muscle mass and strength [15].

On the other hand, several studies have examined the prevalence of sarcopenia in Latin American populations. In a sample of Mexican adults with a mean age of 78.5 ± 7 years, Arango et al. [30] reported a 33.6% prevalence of sarcopenia according to the EWGSOP criteria. Although these authors did not discriminate prevalence by age range or classification on the sarcopenia spectrum, the high frequency may be due to the choice of skeletal muscle mass indicator (calf circumference), which limits the possibility of comparison with the current study. In 91 elderly Brazilians with a mean age of 61.9 ± 8.7 years, Castro et al. [31] reported a 12% prevalence of sarcopenia according to the criteria defined by Chien et al. [32]. In Colombia, a study by González-González et al. [25], using the EWGSOP criteria from 2009, describes a 13.8% prevalence of sarcopenia in 81 women with a mean age of 69.6 ± 3.1 years from the city of Manizales.

The large observed variations in prevalence among studies are because consensus has yet to be reached on the criterion of muscle mass below which sarcopenia should be diagnosed. Also, difference in handgrip strength protocol used (i.e., number of repetitions, hand dominance, dynamometer device, etc.) could explain these discrepancies in prevalence from the studies. Another possible explanation may be the nutritional deterioration to which cancer patients are exposed, mostly based on the involuntary loss of weight, a frequent condition accompanied by reduced intake and systemic inflammation [33]. Within this context, it has been reported that process of muscle mass loss is due to protein degradation mediated by the activation of the ubiquitin-dependent proteasome pathway [34] and, hence, on loss of muscular function [35].

Along with a decrease in muscle size, one of the major contributing factors to the loss of strength and functional capacity is also related with a decrease in muscle quality as a consequence of increased amount of intramyocellular adipose tissue (i.e., muscle fat infiltration) and connective tissue [36,37,38], together with a loss of spinal motoneuron that leads to a decline in in the size and/or number of individual muscle fibers, especially of fast-twitch fibers (i.e., sarcopenia) [39]. Also highlighted is the increase in inflammatory cytokines secreted by the immune system and adipose tissue, such as tumor necrosis factor (TNF)-α or interleukin (IL)-1, or by the tumor itself, i.e., specific catabolic factors of proteolysis-inducing factor-type tumors [40], which activate enzymes that induce protein replacement in skeletal muscle. In a parallel manner, synthesis signals are inhibited, including those produced through anabolic hormones such as insulin-like growth factor 1 and testosterone [41]. Another aspect to consider is the decrease in muscle mass that occurs during the aging process and during cancer, both of which are strongly tied to increased fat mass. Along these lines, Cesari et al. [42] reported that IL-6 and TNF-α were related positively to fat mass and negatively to muscle mass, participating actively in the development of sarcopenia [43,44] by provoking an involuntary loss of muscle mass without initial weight loss. Similarly, high concentrations of IL-6 and TNF-α have been reported in the metabolic states of sarcopenia [45], obesity [46], and SO [47].

With respect to the components of sarcopenia, low values of handgrip strength and skeletal muscle mass index were the most prevalent components, with prevalence rates of 77.6% and 37.8%, respectively, while lower physical performance in the gait speed test had a lower frequency (18.4%). These modifications significantly alter the protein deposits as much as muscle functionality; accordingly, several works report that sarcopenia is the primary cause of fragility in elderly adults [48].

This work showed that 16.3% of the women evaluated had SO, with the highest frequency (40.9%) occurring in the group of women with sarcopenia. From a clinical perspective, changes in body composition taking place in cancer patients indicate the possible combination of low muscle mass and high fat mass, which triggers SO due to the co-existence of excess adiposity and sarcopenia [45]. In this regard, different studies [49,50] show that up to 60% of patients with breast cancer gain weight, and this increase is associated with adverse results, such as diseases associated with obesity. In addition, deregulation of adipocytokines secreted by adipose tissue and by cells under oxidative stress act in synergy on the metabolic anomalies related to obesity [45]. Positive energy balance due to reduced energy expenditure at rest and reduced exercise during treatment could also cause an increase in fat mass, particularly of central fat mass, and thereby produce weight gain [51].

In many cases of SO, a state of insulin resistance facilitates the onset of cardiovascular diseases, such as ischemic heart disease, cerebrovascular disease, and essential arterial hypertension, whose etiology is related to insulin resistance syndrome [52]. In this sense, SO, represented by increased adiposity, has been associated with a defect in the insulin signaling pathway at a post-receptor level and impaired glucose transport through the insulin-regulated glucose 4 transporter pathway [53].

Although several studies have examined the relationship between evaluation components for sarcopenia diagnosis, the mediating role of body composition variables, for example, the relationship between muscle strength and muscle mass, is still unknown [50,54]. Likewise, our results reveal partial mediation with indicators of adiposity (BMI and WC), while total mediation was observed with the body mass (kg) variable. In this regard, it has been confirmed that changes in muscle composition and architecture (e.g., infiltration of intramuscular fat) are related to lower physical performance and function [55]. It is also known that increased adipose tissue, especially in abdominal obesity, induces metabolic disorders, such as insulin resistance, through diverse pathways that imply the involvement of adipocytokines [50,52]. Patients with SO have shown that loss of muscle mass is counterbalanced with increased adipose tissue, an aspect that is not evident when only body weight is measured. Thus, our results reveal the influence of the anthropometric component on muscle mass. Hence, maintaining values >20 kg in muscle strength as measured through dynamometry, along with fat mass values <31.7% and healthy body weight, seems to be a measure that would benefit the muscles as endocrine organs [50,51,52].

Studies in a population of cancer survivors have demonstrated that the parameters of body composition are affected after adjuvant therapies are administered as part of treatment [6,7,8,10]. Other studies evidence that improved muscle mass and diminished adiposity can prevent weight gain and adverse changes in body composition [8,9,50]. Thus, our findings suggest that intervention programs in women who have survived breast cancer must concentrate on the component of muscle strength and control the co-variables of body composition, thereby improving the quality of the skeletal muscle. Longitudinal studies and controlled clinical trials must be performed to investigate in depth the effects of physical exercise on the criteria of physical performance, strength, muscle mass, and inflammatory and cardiometabolic markers [48,56]. An integrated approach will include timely dietary counselling and physical activity. There is evidence to show that complying with a supervised exercise program may help mitigate muscle wasting [57] and sarcopenia [58].

Some aspects must be kept in mind as limitations in this study. For example, the sample size, characteristics of the population, study design, and type of sampling can be considered potential sources of bias. Other variables that can be associated with obesity/sarcopenia were not included, such as ethnicity, nutritional aspects, and levels of physical activity. Another limitation to our study was the use of BIA to assess body composition. EWGSOP reviewed several tools to evaluate body composition and found that dual-energy X-ray absorptiometry and BIA are the most suitable for clinical use [15]. However, no arguments exist to support the believe that the relationships described occur exclusively in the population from which our sample is taken, given that the results were observed to converge with data described in other national and international studies.

## 5. Conclusions

Overall, the results of this research indicate that one in every five women who have survived breast cancer has sarcopenia (i.e., atrophy and muscle performance impairments). These findings seem to emphasize the importance of obesity prevention in women survivors of breast cancer, suggesting that high handgrip strength may not be closely related to greater muscle mass and therefore would not exclude the risk of sarcopenia. Even though it is true that these changes are due to a multifactor process, our findings seem suggest that interventions aimed at optimizing these aspects could be of vital importance to maintaining a healthy body composition in this type of population. Observational studies with larger sample sizes and in other populations and age groups are required, especially longitudinal and prospective studies, to verify the results obtained in this work.

## Figures and Tables

**Table 1:** Diagnostic criteria of sarcopenia according to the European Working Group on Sarcopenia in Older People (EWGSOP).

Stage	Muscle Mass	Muscle Strength		Physical Performance
Pre-sarcopenia	+		and/or	
Sarcopenia	+	+		+
Severe sarcopenia	+	+		+

+: Presence.

**Table 2:** Clinical characteristics of the population women survivors of breast cancer, Bogotá, Colombia.

Characteristics	*n* = 98
Age (Years)	65.53 ± 5.91
Level of schooling *	
Primary	48 (48.9)
High school	43 (43.8)
Professional	7 (7.1)
Socioeconomic level *	
1	9 (9.2)
2	37 (37.8)
3	39 (39.8)
>4	13 (13.2)
Body composition and anthropometry	
Weight (kg)	63.95 ± 11.3
Height (m)	1.51 ± 0.06
Waist circumference (cm)	90.87 ± 12.94
BMI (kg/m^2^)	27.88 ± 4.71
Nutritional classification *	
Normal weight	26 (26.5)
Overweight	38 (38.8)
Obesity	34 (43.7)
Muscle mass (kg)	36.34 ± 5.77
Lean mass (kg)	16.79 ± 2.88
Fat mass (kg)	27.27 ± 7.85
Fat mass (%)	41.51 ± 6.14
Skeletal muscle mass index (absolute muscle mass/size^2^)	7.31 ± 1.07
EWGSOP indicators	
Muscle strength (kg)	16.79 ± 4.98
Gait speed (m/s)	1.22 ± 0.40
Chair stand (rep)	15.6 ± 5.83
Equilibrium (s)	9.15 ± 3.08
Sarcopenia prevalence *	22 (22.4)
Sarcopenic obesity prevalence *	16 (16.3)

Data are shown as mean and ± standard deviation. * These variables are expressed in absolute frequencies and percentages.

**Table 3:** Distribution of body composition variables according to the sarcopenia stages from the EWGSOP.

*n* = 98	Healthy *n* = 22	Risk of Sarcopenia ^†^ *n* = 41	Presarcopenia *n* = 6	Sarcopenia *n* = 22	Severe Sarcopenia *n* = 7	*p* for Trend
Age (Years)	64.27 ± 5.45	65.96 ± 5.94	64.17 ± 5.19	67.27 ± 6.43	66.29 ± 6.10	0.502
Body composition and anthropometry						
Weight (kg)	70.37 ± 14.31	66.41 ± 8.5	59.35 ± 8.46 *	56.21 ± 14.21 *	56.51 ± 6.20 *	<0.001
Height (m)	1.52 ± 0.04	1.49 ± 0.04	1.56 ± 0.05	1.53 ± 0.06	1.48 ± 0.06	0.024
Waist circumference (cm)	96.05 ± 12.14	93.22 ± 10.01	81.67 ± 10.72 *	84.08 ± 10.75 *	89.94 ± 25.45	0.005
BMI (kg/m^2^)	30.10 ± 5.63	29.59 ± 3.72	24.30 ± 3.06 *	24.19 ± 2.81 *	25.46 ± 2.90 *	<0.001
Normal weight, (%) **	4 (18.2)	2 (4.9)	3 (50.0)	14 (63.6)	3 (42.9)	0.149
Overweight *n*, (%) **	7 (31.8)	20 (48.8)	3 (50.0)	6 (27.3)	1 (14.3)	
Obesity *n*, (%) **	11 (50.0)	19 (46.3)	0 (0.0)	2 (9.1)	3 (42.9)	
Muscle mass (kg)	36.45 ± 10.59	37.62 ± 3.95	33.71 ± 10.35	32.84 ± 4.28	32.21 ± 2.82	0.052
Lean mass (kg)	18.29 ± 3.43	17.59 ± 2.68	16.19 ± 1.89	14.79 ± 1.56 *	13.88 ± 1.64 *	<0.001
Fat mass (kg)	30.30 ± 9.37	28.34 ± 7.29	23.20 ± 3.88 *	22.27 ± 6.17	24.29 ± 4.55	0.002
Fat mass (%)	42.94 ± 5.60	42.13 ± 11.48	37.80 ± 2.86	38.63 ± 5.73	42.71 ± 4.23	0.100
Skeletal muscle mass index (absolute muscle mass/size^2^)	7.86 ± 1.39	7.79 ± 2.07	6.58 ± 0.73 *	6.27 ± 0.32 *	6.33 ± 0.49 *	<0.001
EWGSOP indicators						
Muscle strength (kg)	23.43 ± 2.67	15.18 ± 3.43 *	21.12 ± 1.79	13.99 ± 3.91 *	13.21 ± 5.16 *	<0.001
Gait speed (m/s)	1.19 ± 0.35	1.29 ± 1.64	1.37 ± 0.24	1.24 ± 0.33	0.79 ± 0.07 *	0.027
Chair stand (rep)	16.0 ± 3.67	16.4 ± 5.79	15.8 ± 2.29	13.5 ± 2.53 *	13.4 ± 2.72 *	<0.001
Equilibrium (s)	9.40 ± 1.5	9.20 ± 2.0	9.15 ± 1.18	9.13 ± 1.35	9.08 ± 1.14	0.462
Sarcopenic obesity *n*, (%) **	0 (0.0)	4 (18.2)	1 (16.7)	9 (40.9) *	2 (28.5)	0.023

* *p* < 0.05 expresses significant differences with the reference group (normal) through one-way analysis of variance (ANOVA) test, and the multiple comparisons with Bonferroni test. ** These variables are expressed in absolute frequencies and percentages. ^†^ We have used <20 kg in the handgrip strength test as a threshold for risk of sarcopenia.

## Abbreviations

The following abbreviations are used in this manuscript:

ANOVA	Analysis of variance
BIA	Bioelectrical impedance analysis
BMI	Body mass index
EWGSOP	European Working Group on Sarcopenia in Older People
GLOBOCAN project	International Agency for Research on Cancer
IL-1	Interleukin (IL)-1
SD	Standard deviation
SIMMON	Integrated Synergies to Improve Oncological Management in Colombia
SO	Sarcopenic obesity
TNF-α	Tumor necrosis factor alpha
WC	Waist circumference
WHO	World Health Organization
